# Comparison of the Efficacy of Opioid-Free Anesthesia With Conventional Opioid-Based Anesthesia for Nasal Surgeries - A Prospective Randomized Parallel Arm Triple-Blinded Study

**DOI:** 10.7759/cureus.42409

**Published:** 2023-07-24

**Authors:** Hariharan S S, Arul M Ramasamy, Aruna Parameswari, Rajesh Kumar Kodali V, Mahesh Vakamudi

**Affiliations:** 1 Anesthesiology, Sri Ramachandra Institute of Higher Education and Research, Chennai, IND

**Keywords:** decreasing ponv, vas pain scores, dexmedetomidine, functional endoscopic sinus surgery, septoplasty, opioid free anaesthesia

## Abstract

Introduction

In the setting of nasal surgeries, the use of opioid-free anesthesia involving the use of dexmedetomidine, and lignocaine is being investigated as a potential alternative to opioids. This combination of drugs provides sympatholysis, pain relief, and sedative properties, thereby aiming at reducing the negative effects commonly associated with opioid usage. The objective of this study is to evaluate and compare the effectiveness of opioid-free anesthesia using dexmedetomidine and lignocaine versus conventional opioid anesthesia with fentanyl for nasal surgeries. The comparison will be based on the primary outcome of postoperative visual analog scale (VAS) scores. Secondary outcomes assessed were the amount of rescue analgesic consumption, intraoperative sevoflurane usage, intraoperative blood loss, hemodynamic stability, postoperative nausea and vomiting (PONV) scores, and postoperative Ramsay Sedation Scores.

Methods

A triple-blind, prospective, randomized, parallel arm study in which 48 patients planned for elective nasal surgery were allocated randomly to one of two groups. In the study, the population labeled as Group D, comprising 24 participants, received dexmedetomidine at a dosage of 1 mcg.kg^-1^ via intravenous infusion lasting for a duration of 10 minutes prior to the induction of anesthesia. This was followed by a continuous infusion of 0.6 mcg.kg^-1^ h^-1^ throughout the intraoperative period, and intravenous Lignocaine 1.5 mg.kg^-1^ was administered three minutes prior to induction, subsequently an intraoperative infusion of 1.5 mg.kg^-1^ h^-1^. In Group F, consisting of 24 participants, intravenous fentanyl of 2 mcg.kg^-1^ was administered three minutes before the induction. This was subsequently followed by a fentanyl infusion of 0.5 mcg.kg^-1^h^-1 ^in the intraoperative period.

Results

The study findings indicate that Group D had considerably lower postoperative VAS scores from 30 minutes to two hours compared to Group F (p<0.05). The utilization of sevoflurane during the intraoperative period was comparatively reduced in Group D in order to achieve the desired bispectral index (BIS) range of 40-60 (p<0.01). Mean intraoperative blood loss was also lower in Group D (85 ml) compared to Group F (115 ml )(p<0.01). Additionally, Group D had significantly lower rescue analgesic consumption and lower incidence of PONV up to 60 minutes compared to Group F (P-value <0.01). A statistically significant difference was observed between Group D and Group F in terms of lower mean values of both mean arterial pressure (MAP) and heart rate in Group D (p<0.01). The results indicate that the postoperative sedation scores within the first two hours were significantly greater in Group D compared to Group F (p<0.01).

Conclusion

The usage of opioid-free anesthesia has been found to be superior to a traditional opioid-based approach in various aspects, including the provision of sufficient pain relief after surgery, maintenance of stable hemodynamics during the operation, and reduction in occurrences of postoperative nausea and vomiting.

## Introduction

Opioids are frequently employed during the perioperative period as part of a comprehensive anesthesia regimen [1]. Nevertheless, there is a substantial amount of variability among patients in their response to opioids. The administration of opioids is correlated with substantial adverse effects, including respiratory depression after surgery, hyperalgesia after surgery, increased use of pain medication, postoperative nausea and vomiting (PONV), prolonged sedation, ileus, and urinary retention. These consequences can impede the recovery process, delay discharge, or result in unexpected hospital readmission [2,3]. The available evidence [4] suggests that opioids should be utilized judiciously due to their established adverse consequences in the short term, which have the potential to impact both patient-relevant outcomes and expenses. Recent studies [5-7] have demonstrated a correlation between the utilization of opioid-free anesthesia (OFA) in the context of bariatric surgery and accelerated postoperative recovery, reduced length of hospital stay, leading to diminished healthcare costs, early mobilization of patients, and a decline in the negative effects associated with opioid usage. While there have been several studies [5,8]^ ^conducted on OFA in the context of bariatric surgery, the available literature pertaining to OFA in nasal surgery remains scarce, with only a limited number of studies addressing this specific area. Therefore, we conducted a prospective, triple-blind, randomized, parallel-arm study to compare the effect of OFA using intravenous dexmedetomidine and lidocaine with conventional opioid technique using intravenous fentanyl on postoperative pain intensity in patients undergoing nasal surgeries. The primary outcome assessed in our study was postoperative pain scores for 24 hours. The amount of intraoperative sevoflurane consumption, intraoperative hemodynamic alterations in the form of changes in heart rate, systolic blood pressure (SBP), diastolic blood pressure (DBP), mean arterial pressures (MAP), postoperative rescue analgesic consumption, postoperative nausea and vomiting (PONV), and postoperative sedation using Ramsay Sedation Scores were evaluated as secondary outcomes.

## Materials and methods

**Figure 1:**
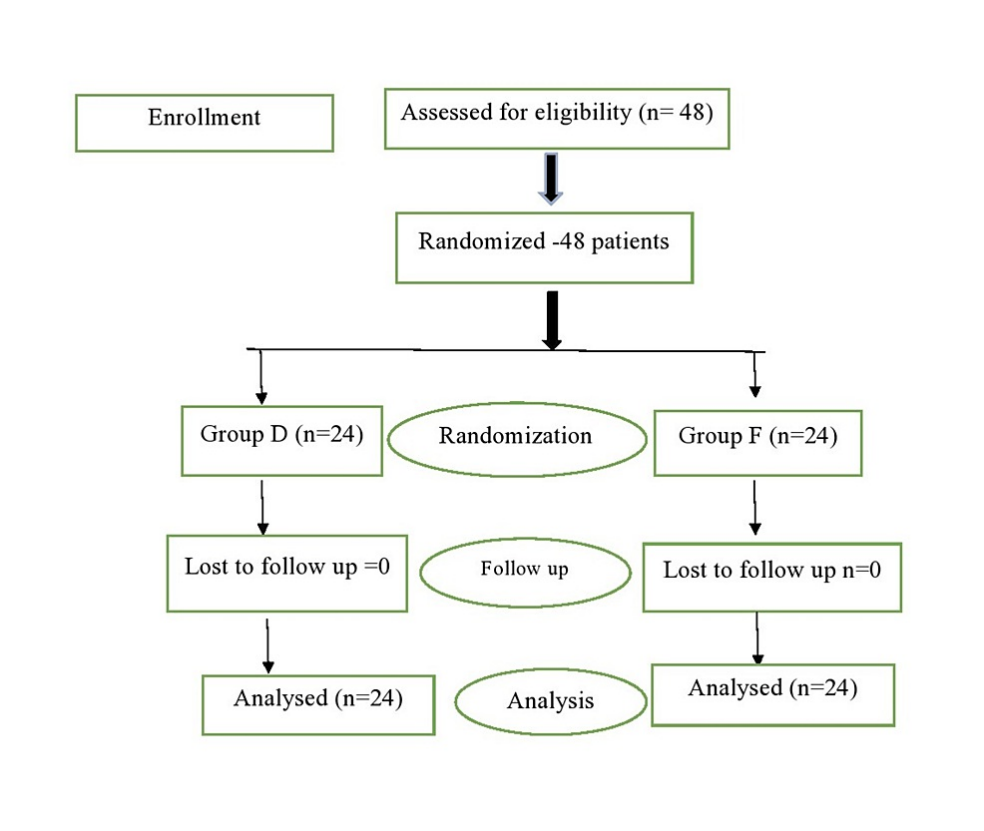
Consolidated Standards of Reporting Trials (CONSORT) diagram showing the process flow

The study enrolled individuals between the ages of 18 and 60 who had an American Society of Anesthesiologists (ASA) physical status of I or II and were scheduled to undergo elective nasal surgeries, including functional endoscopic sinus surgery (FESS) and septoplasty. The study excluded individuals with an ASA physical status of III or above, those with multiorgan failure and unstable hemodynamics prior to surgery, those scheduled for emergency surgery, pregnant and lactating women, asthmatics, and individuals utilizing preoperative alpha-blockers. Prior to the beginning of the clinical trial, individuals who fulfilled the inclusion criteria were asked to give written consent that was both informed and voluntary. Additionally, the study received approval from both the Clinical Trial Registry of our nation and the Institutional Ethics Committee of the hospital (CTRI/2022/01/039288 and IEC/20/OCT/159/39, respectively). The study's participants were allocated randomly to one of two parallel groups. Group F was administered conventional anesthesia that included opioids, which served as the active control. On the other hand, Group D was subjected to opioid-free anesthesia, which was the experimental intervention (Figure 1).

In this study, the participant, anesthesiologist, and outcome assessor were all blinded. The anesthesia provider was unaware of which drug was contained in the syringe and administered the drug according to the research protocol. Loading drugs A and B were administered 10 and three minutes before induction, respectively, and infusion drugs A and B were administered following induction in both of the groups. The study medications were loaded by an anesthesiologist who was independent and not involved in the study or the administration of anesthesia. Another anesthesiologist, also unaware of group assignment or medication loading, collected data and assessed outcomes. In Group F, loading medication A was 0.9% normal saline added in a 50 ml syringe and administered 10 minutes prior to induction, whereas loading medication B was intravenous fentanyl diluted to 10 mcg per ml in a 20 ml syringe and given as a bolus at a dose of 2 mcg.kg^-1^ three minutes prior to induction. In Group D, the drug A used for loading was dexmedetomidine, which was loaded into a 50 mL syringe. It was administered at a dose of 1 mcg.kg^-1^ over a duration of 10 minutes prior to the induction procedure. The administration process involves loading drug B, specifically intravenous lignocaine, into a 20 mL syringe. The dose of lignocaine is calculated at 1.5 mg per kilogram of body weight. This dose is administered as a bolus three minutes prior to the induction. In Group F, infusion medication A was 0.9% normal saline placed into a 50 mL syringe and infused, whereas infusion drug B was intravenous fentanyl loaded into a 20 mL syringe and administered at a rate of 0.5 mcg.kg^-1^h^-1^. In Group D, dexmedetomidine was administered as an infusion medication A through a 50 mL syringe at a dose of 0.6 mcg.kg^-1^h^-1^. Additionally, intravenous lignocaine was administered as infusion drug B during the surgery through a 20 ml syringe at a dose of 1.5 mg.kg^-1^ h^-1^. Prior to induction, an electrocardiogram, oxygen saturation, and non-invasive blood pressure were connected, and baseline values were recorded. After three minutes of ventilation with 100% oxygen and 1% sevoflurane, laryngoscopy and tracheal intubation were performed. In accordance with our department's standard protocol, both groups received a bilateral sphenopalatine block (1.5 mL of 0.5% bupivacaine) prior to the beginning of the procedure. The administration of sevoflurane, oxygen, and air was used to keep the bispectral index within the range of 40 to 60. All participants received 0.1 mg.kg-1 of dexamethasone before the beginning of the surgical procedure. Both groups received IV ondansetron 4 mg and IV paracetamol 1 g 30 minutes prior to extubation. All drug infusions related to the study were ceased ten minutes before the reversal of neuromuscular blockade. The reversal of neuromuscular blockade was accomplished by administering neostigmine intravenously at a dosage of 0.05 mg per kilogram of body weight, along with glycopyrrolate at a dosage of 10 micrograms per kilogram of body weight. After sufficient reversal and oral suctioning, the patients were extubated. If the VAS score was greater than three in the postoperative period, 30 mg IV ketorolac was administered as rescue analgesia. Intraoperative bradycardia (heart rate less than 50 per minute) was treated with 0.2 mg of intravenous glycopyrrolate, and tachycardia (heart rate more than 30% above baseline) during the surgery was treated with titrated doses of intravenous propofol 20 mg doses. Any intraoperative hypotension (mean arterial pressure less than 50) was managed with intravenous ephedrine 6 mg bolus doses. Intraoperative hypertension (mean arterial pressure more than 100) was managed with titrated doses of IV propofol 20 mg doses. All the adverse effects are managed by the concerned anesthesiologist (who was not a participant in the study). Intraoperatively all patients were monitored with standard ASA parameters like heart rate, respiratory rate, mean arterial pressures, and oxygen saturation. We also recorded intra-operative blood loss, amount of sevoflurane consumed, postoperative nausea and vomiting score, postoperative pain scores, and postoperative sedation using a modified Ramsay scale. We determined the sample size with a power of 90% and alpha error of 5% by referencing the mean difference in 24-hour postoperative Visual Analog Scale (VAS) scores between the group that received opioid general anesthesia and the group that received opioid-free anesthesia in a previous study conducted by Mohit Gupta et al. [9].

The statistical analysis was conducted using SPSS software, version 23 (IBM Inc., Armonk, New York). The categorical variables were evaluated using frequency analysis and percentage analysis. The assessment of continuous variables that follow a normal distribution was done by using the calculation of the mean and standard deviation. Conversely, for continuous variables that do not follow a normal distribution, the evaluation was performed by calculation of the median and interquartile range. The Shapiro-Wilk test was used for the assessment of normality. The study employed an unpaired sampled t-test to assess normally distributed continuous variables. The Mann-Whitney U test was employed to assess continuous variables that did not conform to a normal distribution, whereas the Chi-squared test was utilized to evaluate categorical data. Fischer's exact test was used for categorical variables if the expected frequency was less than five in the 2×2 table. All of the aforementioned statistical methods with a probability value below 0.05 are considered as statistically significant.

## Results

The demographic data examined, which included age, BMI, gender, type of procedure, and length of surgery, did not show a statistically significant difference between the two groups (Table 1).

**Table 1:** Comparison of preoperative demographic data between both groups FESS - functional endoscopic sinus surgery, ASA - American Society of Anaesthesiologists Unpaired t-test and Chi-squared test were used to compare both groups.

Variables	Group D	Group F	p-value
Age in years (mean ± SD)	35.0 ± 13.0	34.9 ± 13.8	0.98
Male gender (%)	12 (50%)	12 (50%)	1.0
Type of surgery FESS/ FESS with septoplasty	17/7	20/4	0.49
ASA physical status grade I/ grade II	15/9	15/9	1.0
The total duration of surgery in minutes (mean ± SD)	63.5 ± 9.8	59.6 ± 10.3	0.18
BMI (mean ± SD)	23.1 ± 2.4	22.7 ± 3.4	0.70

We found that Group D had significantly lower mean values for intraoperative blood loss, intraoperative sevoflurane dial setting, and intraoperative sevoflurane consumption compared to Group F (p<0.01) (Table 2).

**Table 2:** Intraoperative and postoperative data comparison between both groups Unpaired t-test was used for the comparison of both groups. *Indicates p-value is significant and it is less than 0.05

Variables (mean ± SD)	Group D	Group F	p-value
Mean Intraoperative blood loss in ml	85.2 ± 19.0	112.3 ± 29.8	<0.01*
Mean intraoperative sevoflurane dial setting	1.6 ± 0.1	2.1 ± 0.1	<0.01*
Total amount of sevoflurane used in ml	15.6 ± 2.0	18.6 3.5	<0.01*
Total amount of mean rescue analgesic (ketorolac) consumption in mg	0 ± 0	15 ± 15.3	<0.01*

The sedation scores of Group D were substantially greater than those of Group F for up to two hours following surgery, as indicated by statistical significance ranging from p<0.01 to p=0.03 (Table 3).

**Table 3:** Comparison of postoperative sedation scores between both groups Unpaired t-test was used for the comparison of both groups. *Indicates p-value is significant and it is less than 0.05

Ramsay sedation score (mean ± SD)	Group D	Group F	p-value
30 min	3.0 ± 0.4	1.7 ± 0.5	<0.01*
60 min	2.7 ±0.6	1.5 ± 0.5	<0.01*
90 min	1.7 ±0.5	1.0 ±0.0	<0.01*
2 hours	1.2 ± 0.4	1.0 ± 0.0	0.03*
4 hours	1.0 ± 0.0	1.0 ± 0.0	1.0
8 hours	1.0 ± 0.0	1.0 ± 0.0	1.0

The PONV scores of Group D were significantly lower than those of Group F up to 60 minutes (p<0.01) (Table 4).

**Table 4:** Postoperative nausea vomiting (PONV) score comparison between both groups Chi-squared test was used for the comparison of both groups. *Indicates p-value is significant and it is less than 0.05

PONV score	Group D	Group F	p-value
At 30 min 0/I/II	24/0/0	9/9/6	<0.01*
At 60 min 0/I/II	18/6/0	1/8/15	<0.01*
At 90 min 0/I/II	21/3/0	15/8/1	0.11

The pain scores within Group D were observed to be significantly lower than those in Group F for up to two hours after the surgery, with a statistical significance of p<0.01 to 0.01 (Table 5).

**Table 5:** Postoperative pain score comparison between both groups Mann-Whitney U test was used to compare the pain scores. *Indicates p-value is significant and it is less than 0.05

Postoperative pain score, median (IQR)	Group D	Group F	p-value
At 30 min	0 (0-0)	2 (1-3)	<0.01*
At 60 min	0 (0-1)	3 (2-4)	<0.01*
At 90 min	2 (2-2)	3 (2-3)	0.01*
At 2 hours	2 (1-2)	2 (2-3)	0.01*
At 4 hours	1 (1-1)	1 (1-1)	0.45
At 6 hours	1 (1-1)	1 (1-1)	0.46
At 12 hours	0 (0-0)	0 (0-0)	0.81
At 24 hours	0 (0-0)	0 (0-0)	0.99

**Figure 2:**
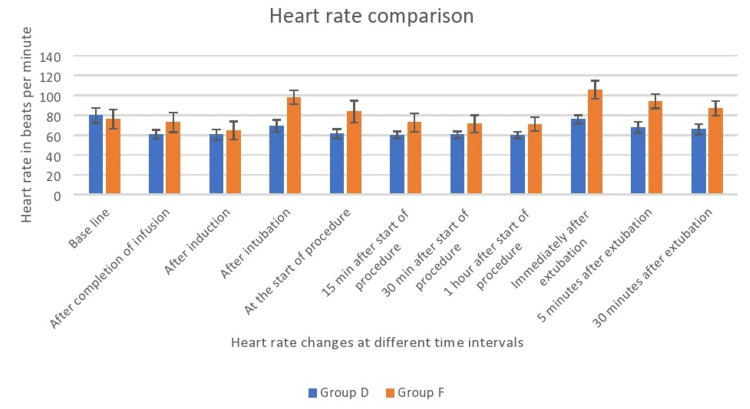
Heart rate comparison between both groups Data are represented as mean with error bars showing standard deviation.

In Group F, 12 out of 24 patients required rescue analgesia within one hour, and the mean rescue analgesic ketorolac consumption was 15 mg which was significant when compared to Group D (Table 2). The findings indicate a statistically significant decrease in heart rate among subjects in Group D from the time of infusion completion to 30 minutes following extubation (p<0.01; Figure 2).

**Figure 3:**
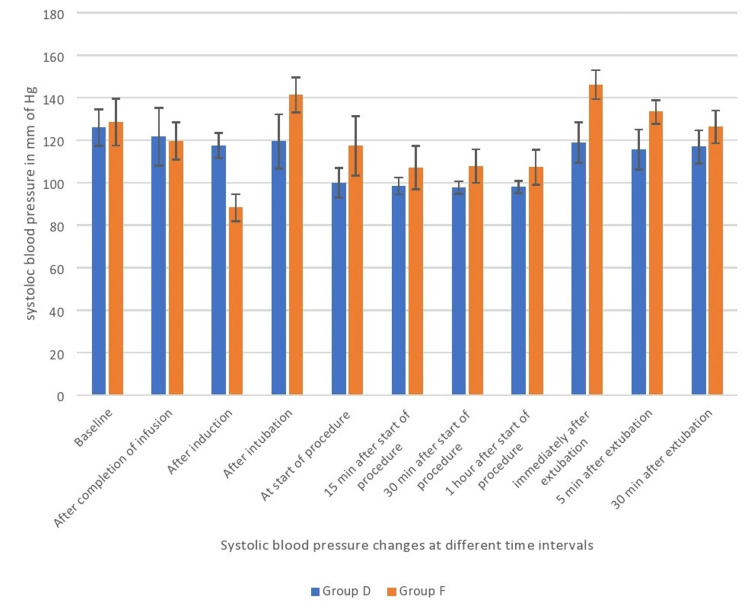
Systolic blood pressure comparison between both groups Data are shown as mean systolic blood pressure with error bars showing standard deviation.

The systolic blood pressure (SBP) in Group D showed a statistically significant reduction compared to Group F from the period following intubation to 30 minutes after extubation (p<0.01; Figure 3).

**Figure 4:**
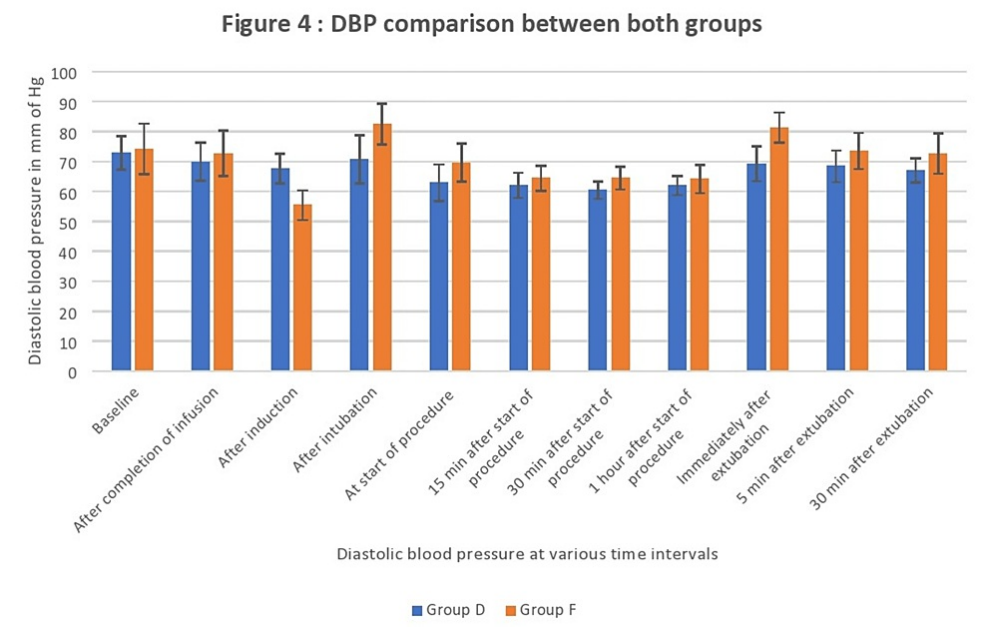
Diastolic blood pressure comparison between both groups Data are shown as mean diastolic blood pressure with error bars showing standard deviation.

From intubation to 30 minutes after extubation, the diastolic blood pressure (DBP) in Group D was substantially lower than in Group F (p=0.04 to p<0.01; Figure 4).

**Figure 5:**
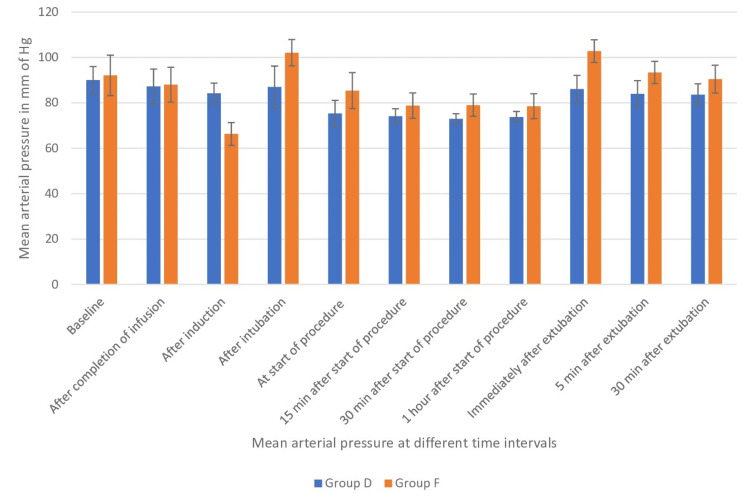
Mean arterial pressure comparison between both groups Data shown as mean of mean arterial pressure with error bars showing standard deviation.

Group D had substantially lower mean arterial pressures than Group F from intubation to 30 minutes after extubation (p<0.01; Figure 5).

Throughout the study period, oxygen saturation was comparable in both groups. When compared to one patient in Group D, four patients in Group F experienced hypotension. Sixteen patients in Group F had hypertension, compared to a single patient in Group D. In Group F, eight patients showed tachycardia. Two patients in Group D had bradycardia (Table 6).

**Table 6:** Intraoperative adverse events comparison between both groups Fischer's exact test was used for the comparison of both groups. * indicates p-value is less than 0.05 and it is significant

Adverse event	Group D (N=24)	Group F (N=24)	p-value
Hypotension (n)	1	4	0.34
Hypertension (n)	1	16	<0.01*
Bradycardia (n)	2	0	0.48
Tachycardia (n)	0	8	0.04*

.

## Discussion

In our study, we compared conventional opioid anesthesia with opioid-free anesthesia with dexmedetomidine and lignocaine. The primary outcome assessed was a postoperative pain score comparison between both groups for 24 hours. We also assessed secondary outcomes like intraoperative sevoflurane consumption, intraoperative blood loss, hemodynamic changes, postoperative rescue analgesic consumption, postoperative sedation scores, and PONV scores between both groups.

In our study, all demographic variables like gender, BMI, age, type of surgery, ASA physical status, and surgery duration were comparable between the two groups (Table 1). The study revealed that participants belonging to Group D reported significantly lower levels of pain up to two hours post-surgery in comparison to those in Group F. Mulier et al. in their study found that the group of patients who did not receive opioids had significantly lower postoperative VAS scores compared to the group who received opioids during anesthesia. According to Gupta and colleagues [9], the postoperative Visual Analog Scale (VAS) scores were lower in the group that received opioid-free anesthesia compared to the group that received opioids. Ahmed and colleagues [10]^ ^have reported that the group receiving opioid-free anesthesia exhibited lower postoperative pain scores in comparison to the control group. According to a meta-analysis conducted by Grape et al. [11], it was found that the postoperative pain scores were comparatively lower in the dexmedetomidine group as compared to the remifentanil group. According to a meta-analysis conducted by Singh et al. [12], it was found that the dexmedetomidine group had lower perioperative pain scores compared to the group receiving conventional analgesia. Decreased pain scores in the opioid-free anesthesia group were explained by the analgesic and antinociceptive properties of dexmedetomidine. In contrast to the present study's results, Choi et al. [13]. reported a lack of statistically significant differentiation in pain scores between the opioid-free and opioid-administered groups. One possible explanation for the observed outcome could be the administration of a lower dose of dexmedetomidine. However, it is important to note that our study utilized a combination of dexmedetomidine and lidocaine for pain management.

We found that the opioid-free anesthesia group required significantly less sevoflurane during surgery to maintain a bispectral index (BIS) range of 40-60, with a mean of 1.6%, compared to the opioid group, which had a mean of 2.1%. It was observed that the overall quantity of sevoflurane utilized was comparatively lower in the opioid-free anesthesia group as opposed to the opioid group (Table 2). This may be due to the effect of dexmedetomidine on BIS, leading to a reduction of sevoflurane requirement to achieve target BIS 40-60. In contrast, opioids don't have any effect on BIS, leading to increased sevoflurane consumption. Similarly, Patel et al. [14] also reported that dexmedetomidine use along with sevoflurane reduces the end-tidal sevoflurane at five minutes post-intubation and 60 min post-intubation. The study conducted by Feld et al. [15] showed that the average end-tidal desflurane concentration was comparatively lower in the dexmedetomidine group, and it was similar to the opioid group. The lower dose of dexmedetomidine used for infusion may have contributed to this outcome; however, in our study period, we employed both dexmedetomidine and lignocaine for infusion. The findings of our study show that the amount of blood loss during surgery was notably reduced in Group D when compared to Group F. This may be due to lower mean arterial pressures in Group D due to dexmedetomidine infusion. Similarly, Ayoglu et al. [16] reported a significant reduction in blood loss during septoplasty procedures in the group administered with dexmedetomidine compared to the control group. Group D had higher postoperative sedation scores up to 90 minutes than Group F. This may be due to the fact that dexmedetomidine and lignocaine cause a longer period of sedation than opioids, but unlike opioids, this sedation did not induce respiratory depression. Similarly, Karabayili et al. [17] reported that the dexmedetomidine group had higher sedation scores than the remifentanil group. In our study, Group D had considerably lower PONV scores than Group F. This is likely due to the elimination of intraoperative opioid use and the decreased intraoperative sevoflurane requirement.

Mulier et al. [18] and Elsaye et al. [19] have previously conducted research indicating that the occurrence of postoperative nausea and vomiting (PONV) was less frequent in the group that did not receive opioids compared to the group that did receive opioids. According to Ziemann-Gimmel et al. [20], the implementation of opioid-free total intravenous anesthesia (TIVA) in bariatric surgery patients results in a 17.3 percent decrease in the incidence of postoperative nausea and vomiting (PONV) when compared to the use of general anesthesia with volatile anesthetics and opioids. Our study revealed that Group F exhibited greater hemodynamic fluctuations, specifically exceeding a 20% change in heart rate and mean arterial pressures, in comparison to Group D. The present study revealed a statistically significant decrease in heart rates in Group D as compared to Group F during the period from completion of infusion to 30 minutes after extubation. A previous study by Jain et al. [21] also reported lesser heart rates in the dexmedetomidine group than in the fentanyl group (Figure 2). We also observed lower systolic pressures, mean arterial pressures, and diastolic pressures in Group F after induction than in Group D. This may be due to the initial hypertensive response of dexmedetomidine as described by Kaur et al. [22], which resulted in lesser fall in pressures with propofol induction, whereas in opioid group vasodilatation with propofol resulted in fall in blood pressures (Figures 3, 4, 5). In our study, we found that Group D exhibited a significant decrease in systolic pressures, mean arterial pressures, and diastolic pressures from the beginning of the procedure to 30 minutes after extubation. Similarly, Vaswani et al. [23] found that the dexmedetomidine group had lower systolic, mean arterial, and diastolic pressures compared to the fentanyl group. Additionally, the dexmedetomidine group showed better hemodynamic stability than the fentanyl group. In contrast to our study, Mulier et al. reported no significant difference in hemodynamic changes between the opioid group and the opioid-free anesthesia group. This may be due to the use of sufentanil, which maintained myocardial stability and resulted in a lesser change in hemodynamics in the intraoperative period [18].

Limitations

In our study, we monitored operative nausea and vomiting for 90 minutes in the post-anesthesia care unit (PACU). We also didn't record postoperative sedation scores beyond eight hours in the postoperative period. We also didn't compare opioid-free anesthesia with dexmedetomidine alone versus dexmedetomidine with lignocaine with conventional opioid anesthesia. A larger sample size may be needed for this study. In our study, agitation scores and surgical field quality assessment with the Wormald grading scale were not done. 

## Conclusions

The results of this study indicate that the group receiving OFA experienced superior analgesic effects in the initial hours compared to the group receiving opioid anesthesia. The administration of anesthesia without opioids was associated with decreased PONV scores, decreased rescue analgesic consumption, enhanced sedation, and stable hemodynamics throughout the duration of the study.
